# Loss of miR-107, miR-181c and miR-29a-3p Promote Activation of Notch2 Signaling in Pediatric High-Grade Gliomas (pHGGs)

**DOI:** 10.3390/ijms18122742

**Published:** 2017-12-17

**Authors:** Giuseppina Catanzaro, Claudia Sabato, Michele Russo, Alessandro Rosa, Luana Abballe, Zein Mersini Besharat, Agnese Po, Evelina Miele, Diana Bellavia, Martina Chiacchiarini, Marco Gessi, Giovanna Peruzzi, Maddalena Napolitano, Manila Antonelli, Angela Mastronuzzi, Felice Giangaspero, Franco Locatelli, Isabella Screpanti, Alessandra Vacca, Elisabetta Ferretti

**Affiliations:** 1 Department of Experimental Medicine, Sapienza University, Viale Regina Elena, 291, 00161 Rome, Italy; giuseppina.catanzaro@uniroma1.it (G.C.); luana.abballe@uniroma1.it (L.A.); zeinmersini.besharat@uniroma1.it (Z.M.B.); alessandra.vacca@uniroma1.it (A.V.); 2 Department of Molecular Medicine, Sapienza University, 00161 Rome, Italy; claudia.sabato@uniroma1.it (C.S.); michele-russo@hotmail.it (M.R.); agnese.po@uniroma1.it (A.P.); diana.bellavia@uniroma1.it (D.B.); martina.chiacchiarini@uniroma1.it (M.C.); maddalena.napolitano@uniroma1.it (M.N.); isabella.screpanti@uniroma1.it (I.S.); 3 Center for Life NanoScience@Sapienza, Istituto Italiano di Tecnologia, 00161 Rome, Italy; alessandro.rosa@uniroma1.it (A.R.); Giovanna.Peruzzi@iit.it (G.P.); 4 Department of Biology and Biotechnology “Charles Darwin”, Sapienza University of Rome, Piazzale Aldo Moro 5, 00185 Rome, Italy; 5 Department of Hematology/Oncology and Stem Cell Transplantation, Bambino Gesù Children’s Hospital, Istituto di Ricovero e Cura a Carattere Scientifico, 00165 Rome, Italy; evelina.miele@opbg.net (E.M.); angela.mastronuzzi@opbg.net (A.M.); franco.locatelli@opbg.net (F.L.); 6 Department of Histopathology, Fondazione Policlinico Universitario “A. Gemelli”, Università Cattolica Sacro cuore, Largo A. Gemelli 8, 00168 Rome, Italy; mgessimd@yahoo.com; 7 Department of Radiological, Oncological and Pathological Science, Sapienza University, 00161 Rome, Italy; manila_antonelli@yahoo.it (M.A.); felice.giangaspero@uniroma1.it (F.G.); 8 Istituto di Ricovero e Cura a Carattere Scientifico Neuromed, Pozzilli, 86077 Isernia, Italy; 9 Department of Pediatrics, University of Pavia, 27100 Pavia, Italy; 10 Institute Pasteur-Foundation Cenci Bolognetti, Sapienza University, 00161 Rome, Italy

**Keywords:** pediatric high-grade gliomas, Notch2 signaling, microRNAs, miR-107, miR-181c, miR-29a-3p, cell proliferation

## Abstract

The mechanisms by which microRNAs control pediatric high-grade gliomas (pHGGs) have yet to be fully elucidated. Our studies of patient-derived pHGG tissues and of the pHGG cell line KNS42 revealed down-regulation in these tumors of three microRNAs, specifically miR-107, miR-181c, and miR-29a-3p. This down-regulation increases the proliferation of KNS42 cells by de-repressing expression of the Notch2 receptor (Notch2), a validated target of miR-107 and miR-181c and a putative target of miR-29a-3p. Inhibition (either pharmacologic or genetic) of Notch2 or re-expression of the implicated microRNAs (all three combined but also individually) significantly reduced KNS42 cell proliferation. These findings suggest that Notch2 pathway activation plays a critical role in pHGGs growth and reveal a direct epigenetic mechanism that controls Notch2 expression, which could potentially be targeted by novel forms of therapy for these childhood tumors characterized by high-morbidity and high-mortality.

## 1. Introduction

Gliomas represent the most common brain tumors in children. Approximately 21% of all primary pediatric gliomas are high-grade tumors [1,2]. These tumors are histologically similar to adult high-grade gliomas (aHGGs), but their genetic and epigenetic features reflect intrinsic differences with respect to their adult counterparts. Recent genome and epigenome profiling of pediatric high-grade gliomas (pHGGs) has markedly expanded our knowledge of their biology. Nevertheless, the therapeutic options for these tumors are still very limited, and the long-term outlook for patients is unfavorable, with high levels of both morbidity and mortality [3,4]. Identifying the molecular pathways that drive pHGGs is essential for the development of new, more effective treatment strategies [4,5].

The Notch signaling pathway regulates a number of cellular processes, such as gliogenesis, self-renewal, and cell-fate specification [6,7,8]. The Notch genes encode four transmembrane receptors (Notch1–4), which are activated by their interaction with ligands of the Serrate/Jagged and Delta families. Ligand binding induces proteolytic cleavages of the receptor, which ultimately result in the release of the intracellular domain and its migration into the nucleus, where it activates the transcription of Notch target genes [9,10,11]. Deregulation of the Notch pathway has been described in a variety of cancers [5,12,13,14,15,16], including aHGGs [12,17,18], but little is known about the relevance of Notch signaling in pHGGs. Immunohistochemical studies of patient-derived tumor tissues indicate that active Notch signaling is a feature of pHGGs, including those that are negative for Notch1 receptor (Notch1) [19]. This observation raises the possibility that other Notch receptors may be playing important functional roles in these tumors. Thus far, however, this issue has yet to be explored.

In the present study, we analyzed Notch2 receptor (Notch2) expression in tissue samples of pHGGs and found it to be increased with respect to that found in normal brain tissue. Pharmacological or genetic suppression of Notch2 signaling in the human pHGG cell line, KNS42, significantly reduced pHGG cell proliferation rates. In light of the growing body of evidence pointing to microRNAs as major mediators of deregulated gene expression in tumors [20], including pHGGs [5], we hypothesized that the activation of Notch2 signaling in pHGGs might be related at least in part to under-expression of one or more microRNAs. Here we show that three microRNAs previously reported to be down-regulated in pHGGs [5] were found to target the 3′-UTR of Notch2: miR-107, miR-181c and miR-29a-3p, and their re-expression in KNS42 cells produced substantial reductions in Notch2 protein levels and cell proliferation rates. These findings reveal a novel network that controls pHGG cell growth and identify new molecular targets for more effective treatment of these devastating pediatric brain tumors.

## 2. Results

### 2.1. Notch1 and Notch2 Receptor Expression in pHGG Tumors

**Figure 1:**
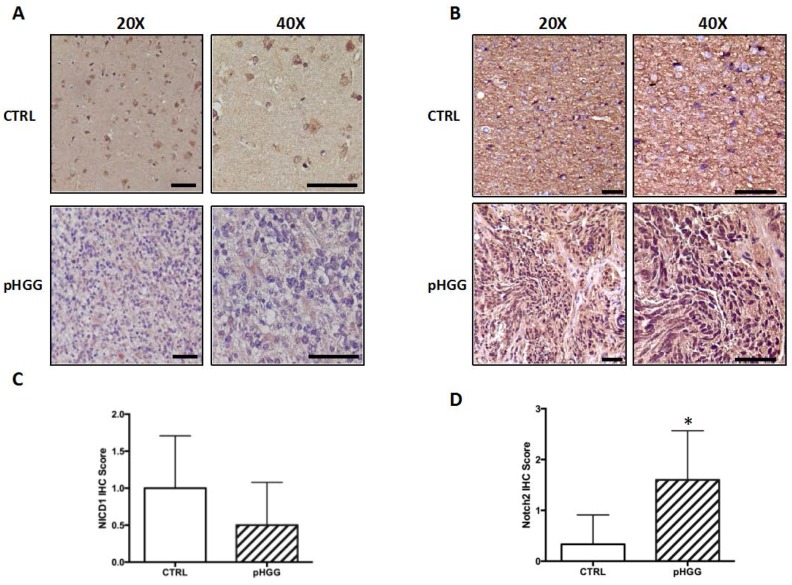
Notch1 and Notch2 expression in pHGG and non-neoplastic brain tissues. Representative images of immunohistochemical (IHC) staining of NICD1 (**A**) and Notch2 (**B**) and relative IHC scores (**C**,**D**) for nuclear expression of NICD1 and Notch2 in 10 pHGGs and normal brain tissue. * *p* < 0.05 vs. control (CTRL). Scale bars in (**A**,**B**): 100 μm.

We assessed nuclear expression levels of the Notch1 and Notch2 in tissue sections from pHGGs. Sections of normal brain tissue were used as reference control (CTRL). As expected, based on data reported by Fouladi et al., the pHGG tissues displayed weak or any nuclear positivity for the activated form of Notch1 (NICD1), which was comparable to that of the control tissue (Figure 1A,C). In contrast, nuclear positivity for Notch2 in the tumor tissues was markedly stronger than that seen in the non-neoplastic brain tissue (Figure 1B,D). These findings indicate that pHGGs are characterized by increased levels of activated Notch2 (NICD2), supporting our hypothesis that this Notch receptor plays a functional role in these tumors. They might also explain the Notch signaling activation observed in pHGGs in which Notch1 expression was low or absent [19].

### 2.2. Notch2 Inhibition Reduces pHGG Cell Proliferation

**Figure 2:**
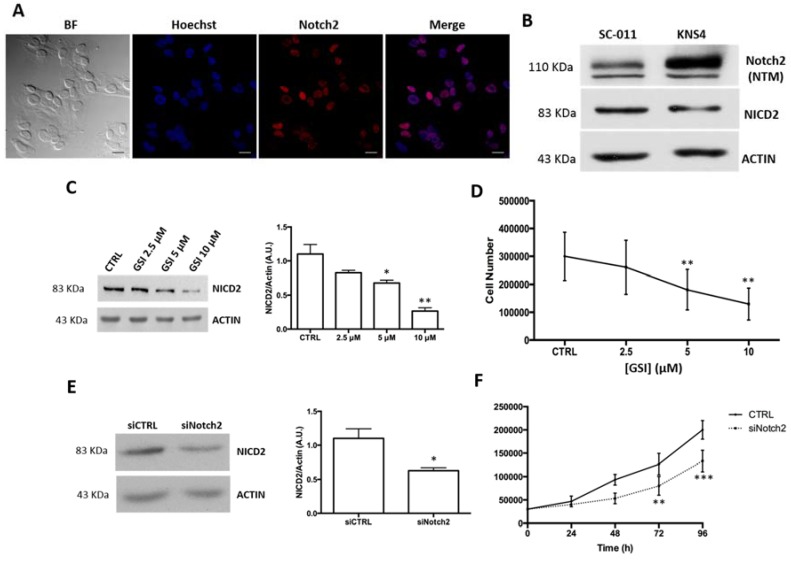
Notch2 expression in KNS42 cells and the impact of its inhibition on proliferation. (**A**) Immunofluorescence labeling of Notch2 expression in KNS42 cells counterstained with the nuclear marker Hoechst. (BF, bright field.) Scale bars: 20 μm; (**B**) Western blot analysis of the trans-membrane form of Notch2 (Notch2 NTM) and NICD2 levels in KNS42 cells and SC-011 cells (used as positive controls); (**C**,**D**) dose-dependent effects of 96 h exposure to GSI on (**C**) NICD2 levels and (**D**) proliferation in KNS42 cells; (**E,F**) Effects of siRNA-mediated knockdown of Notch2 in KNS42 cells; (**E**) Western blot analysis of NICD2 levels 96 h after transfection. (**F**) Time-course of the effects of Notch2 silencing on KNS42 cell proliferation. * *p* < 0.05, ** *p* < 0.01, *** *p* < 0.001 vs. CTRL (untreated cells in panel C and D, silencing negative control-transfected cells in panel E and F).

We next investigated the biological role of Notch2 in pHGGs by using the pHGG cell line KNS42 [21]. As shown in Figure 2A, immunofluorescence analysis showed that these cells—like the patient-derived pHGG tissues examined by IHC—contained high nuclear levels of Notch2. In particular, by counting the number of positive cells nuclei, we obtained that about the 90% of KNS42 expressed Notch2 in the cell nuclei. This finding was confirmed both by Western blot analysis performed with a specific antibody for NICD2 (Figure 2B) and by performing a nuclear/cytoplasm fractionation assay that demonstrates that NICD2 is only expressed in cell nuclei (Figure S1).

The KNS42 cells were then treated for 96 h with the gamma-secretase inhibitor (GSI), *N*-(*N*-(3,5-difluorophenacetyl)-l-alanyl)-2-phenylglycine *t*-Butyl ester [22], which blocks the activity of all four Notch receptors. Western blot studies revealed dose-dependent reduction of NICD2 levels in treated cells (Figure 2C) and this effect was associated with a decline in cell proliferation, which became significant after treatment with GSI both at 5 and 10 μM (Figure 2D). This anti-proliferative effect was due to apoptosis, as demonstrated by the significant increase in the cleaved form of PARP shown in Supplementary Figure S2. To investigate the contribution of Notch2 inhibition to the effects produced by the GSI, we transfected KNS42 cells with a siRNA that specifically targeted Notch2 (siNotch2). We observed appreciably lower levels of NICD2 in transfected cells (Figure 2E) without any reduction of Notch1 expression level, as reported in Supplementary Figure S3, underlying that siNotch2 was specific to Notch2 only. The lower levels of NICD2 were associated with significant decreases in cell proliferation, which were already evident 72 h after siNotch2 silencing (Figure 2F). Collectively, these results confirm our hypothesis that Notch2 plays a substantial role in the Notch-mediated control of pHGG cell proliferation.

### 2.3. Notch2 High Levels Are Maintained by Low Levels of miR-107, miR-181c and miR-29a-3p

Deregulated microRNA expression has been implicated in the cancer-related overexpression of numerous oncogenes, and our group has already shown that multiple microRNAs are differentially expressed in pHGGs [5]. To investigate the mechanism underlying the increased Notch2 expression in pHGG cells, we therefore examined the list of microRNAs that had displayed down-regulated expression in pHGGs (vs. normal brain tissue) in our previous study [5]. Interrogation of miRTarBase and microRNA.org revealed Notch2 to be a validated target of two of the microRNAs on this list (miR-107 and miR-181c) ([23,24,25] and (http://mirtarbase.mbc.nctu.edu.tw) a putative target of a third, miR-29a-3p (microRNA.org)).

**Figure 3:**
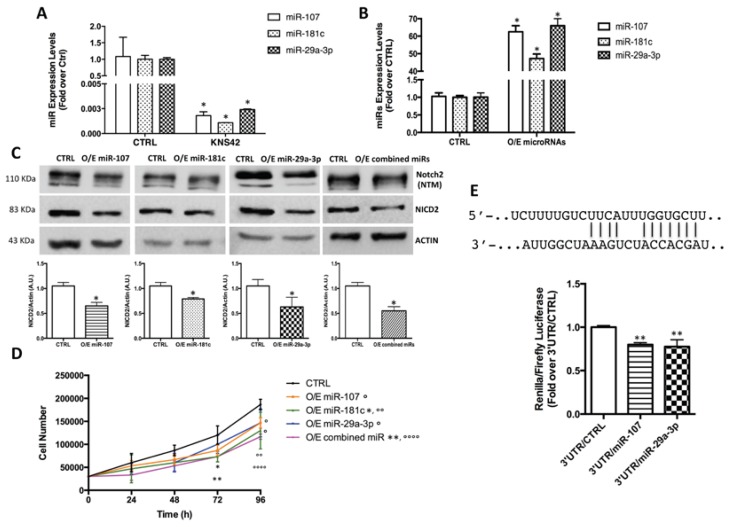
miR-107, miR-181c and miR-29a-3p inhibit pHGG cell proliferation by targeting Notch2. (**A**) Single assay qPCR validation of miR-107, miR-181c, and miR-29a-3p expression in KNS42 cells versus non-neoplastic total brain (CTRL). * *p* < 0.05 vs. CTRL; (**B**,**C**) KNS42 cells were transfected with 20 nM of miR-107, miR-181c, or miR-29a-3p: pre-transfection (CTRL) and 48 h-post-transfection (O/E) levels of (**B**) each microRNA and (**C**) of the trans-membrane form of Notch2 (Notch2 NTM) and of NICD2. * *p* < 0.05 vs. CTRL; (**D**) KNS42 cell proliferation after O/E of the three microRNAs, separately and combined. Significant differences vs. CTRL at 72 h (*p < 0.05, ** *p* < 0.01) and at 96 h (° *p* < 0.05, °° *p* < 0.01, °°°° *p* < 0.0001); (**E**) Renilla activity induced by ectopic expression of Notch2 and negative control (CTRL) in KNS42 cells transfected with Renilla vector bearing the Notch2 3′UTR. miR-107, whose targeting of Notch2 has been previously validated, was used as positive control. Results are expressed as the ratio of Renilla to Firefly luciferase activity. ** *p* < 0.01 vs. 3′UTR/CTRL.

Consistent with our previous findings in pHGGs [5], all three of these microRNAs were expressed at significantly lower levels in KNS42 cells than in non-neoplastic brain tissues (CTRL) (Figure 3A). To analyze the biological impact of this down-regulation, we then overexpressed the microRNAs, individually and combined, in KNS42 cells. After verifying the physiological range of their re-expression levels by RT-qPCR (Figure 3B), we assessed the ability of the microRNAs to diminish NICD2 protein levels. As shown in Figure 3C, both overexpression of each microRNA and of their combination significantly decreased the levels of NICD2. Based on the findings shown in Figure 2, we expected that overexpression of these microRNAs would also be associated with reduced pHGG cell proliferation. Trypan blue exclusion assays showed that KNS42 cell death was not significantly affected by overexpression of any of the microRNAs, even when combined (Data not shown). In contrast, as shown in Figure 3D, by post-transfection hour 72, proliferation was already significantly decreased in cells overexpressing all three microRNAs or miR-181c alone. By 96 h, these reductions were even more evident, and the effect was also statistically significant for cells overexpressing miR-107 or miR-29a-3p alone. Importantly, at both time points, the anti-proliferative effect was more substantial in cells subjected to combined overexpression of the three microRNAs, indicating that miR-29a-3p, miR-107, and miR-181c may act synergically to check KNS42 cell proliferation. We analyzed the role of these three microRNAs in other two immortalized cell lines derived from different glioma grades. Specifically, we used two pediatric low-grade glioma cell lines: Res259, derived from a grade II diffuse astrocytoma, and Res186, derived from a grade I pilocytic astrocytoma. Firstly, we evaluated the level of expression of miR-29a-3p, miR-107, and miR-181c in Res259 and Res186. As shown in Supplementary Figure S4A, we found that their level of expression was comparable to the one we found in KNS42 cells. Then, as reported in Supplementary Figure S4B and S4C, we also demonstrated the ability of these microRNAs in reducing cell proliferation after their re-expression in both pediatric low-grade glioma cell lines. Specifically, Res259 cells seem to be more sensitive than Res186. Cell proliferation indeed was significantly affected by the overexpression of all microRNAs in Res259; while in Res186 only miR-29a-3p and the combined overexpression of the three microRNAs induced a significant effect. The anti-proliferative action of these microRNAs in Res259 and Res186 cells did not increase after 96h in respect to what we observed at 48h (Data not shown).

As noted above, unlike that of miR-107 and miR-181c [23,24,25], the binding of miR-29a-3p to the 3′-UTR of Notch2 has been never experimentally validated. To address this gap, we cloned a portion of the Notch2 3′UTR containing the putative binding site for miR-29a-3p into a luciferase reporter vector and transfected it into KNS42 cells. As shown in Figure 3E, re-expression of miR-29a-3p in these cells significantly reduced expression of the reporter gene in the recombinant vector containing the 3′-UTR of Notch2, thereby providing the first experimental evidence that miR-29a-3p is a direct negative regulator of Notch2 expression. 

Taken together, these observations confirm that the high levels of Notch2 of pHGG cells are maintained at least in part through the down-regulated expression of miR-107, miR-181c, and miR-29a-3p.

## 3. Discussion

MicroRNAs are critical components of the post-transcriptional machinery that regulates tumor cell growth [26,27]. In the present study we identified a microRNA-based mechanism that activates proliferative Notch2 signaling in pHGGs. In particular, our data show that: (1) pHGGs frequently express high levels of NICD2 and little or no Notch1; (2) pharmacological inhibition or siRNA-mediated knockdown of Notch2 in KNS42 pHGG cells significantly reduces their proliferation rates; (3) the hyper-activation of Notch2 signaling in pHGG cells is maintained at least in part by the down-regulated expression of three Notch2-targeting microRNAs—miR-107, miR-181c, and miR-29a-3p—in KNS42 cells.

To the best of our knowledge, to date, only two studies have investigated the roles of Notch signaling in pHGGs. In 2011, Fouladi et al. reported that FFPE sections of grade III and IV malignant gliomas removed from pediatric patients displayed intense nuclear staining for two transcription factors that are downstream effectors of the Notch pathway, HES1 and HES5, and this positivity was also observed in those tumors that were immunonegative for the Notch1 [19]. More recently, Dantas-Barbosa et al. (2015) showed that pediatric glioma xenografts and the pediatric glioma cell line, SF188, express Notch1, the Notch ligand (*DLL1*), and several of the Notch pathway’s downstream target genes (*HES1*, *HEY1*, *MYC* and *FBXW7*), but neither pharmacological nor genetic blockade of Notch1 was capable of reducing pHGG cell growth. Nevertheless, *MYC* resulted to be overexpressed in these pediatric glioma xenografts [28]. Therefore, even though the literature studies are not numerous, both of them report evidence that Notch pathway is active in pHGGs.

The Notch signaling pathway is a highly conserved pathway that plays major roles in many cellular processes. Its function is strongly cell context-dependent [9,29]. Notch signaling can play oncogenic as well as oncosuppressive roles in tumorigenesis, and the four Notch receptors are also characterized by functional diversity, even within the same biological setting [30,31]. 

The present work is the first attempt to delineate the specific role of Notch2 in pHGGs. The increased nuclear levels of NICD2—the active form of the protein—that we documented in pHGGs (patient-derived tissues as well as the KNS42 cell line) support a role for Notch2 signaling in these tumors. This hypothesis is further supported by the results of our experiments with GSI and siNotch2 in KNS42 cells, which showed that the increased activation of Notch2 in pHGG cells enhances their proliferation. An oncogenic role for Notch2 in HGGs had previously been reported only in adult tumors [18,32,33]. 

Our findings also reveal that the Notch2 activation documented in pHGG cells is determined epigenetically, more specifically, by the down-regulated expression of three microRNAs. Two of these (miR-107 and miR-181c) had already been shown to target the Notch2 3’UTR [23,24]. We used a luciferase reporter assay to validate the binding to this region of the third, miR-29a-3p. We investigated the effect of the down-regulation of these microRNAs only on Notch2 however, of note, other genes such as *Bcl2*, *Cdc42*, *CDK6*, *CRKL*, *HMGA2*, *KLF4*, *PLAG1*, *VEGFA*, are validated target for two and putative for one of the microRNAs. Therefore, we cannot exclude that they may affect cell proliferation in the pHGG context. Interestingly, all the three microRNAs have been reported to control proliferation in malignant gliomas in adults [34,35,36,37], but few studies have analyzed microRNA expression in pHGGs. Li et al. [38] found that miR-107 and miR-181c were down-regulated in both high- and low-grade pediatric gliomas. Jha et al. [39] compared microRNA expression levels in pHGGs with those found in brain tissue samples from patients operated for epilepsy. The tumor tissues were characterized by down-regulation of the miR-379/656 cluster members. Up-regulated expression of several microRNAs was also observed, including those belonging to the miR-17/92 cluster, which is consistent with our previous findings [5]. Eguía-Aguilar et al. [40] have reported decreased expression of miR-124-3p, miR-128-1 and miR-221-3p in astrocytomas of all grades relative to levels found in normal brain tissues. More recently, Liang et al. [41] reported the down-regulation of miR-137 and miR-6500-3p in three pediatric glioma cell lines, including two that were derived from high-grade tumors.

In summary, our results document a link between aberrant oncogenic pathway activation mediated by Notch2 and the down-regulated expression of miR-107, miR-181c, and miR-29a-3p and suggest that this network is a key regulator of pHGG cell growth. These findings have potential implications for new targeted therapies for these tumors since the inhibition of abnormal activated pathways could be an effective therapy to overcome the high levels of morbidity and mortality underlying poor long-term outcomes. 

## 4. Materials and Methods

Unless otherwise stated, commercially available products were used according to the manufacturer’s instructions/protocols.

### 4.1. Ethics Statement

The study was conducted in accordance with the 1964 Declaration of Helsinki and its later amendments or comparable ethical standards and with the guidelines of the ethical policies of the involved institutions.

### 4.2. Histology

Formalin-fixed paraffin-embedded (FFPE) samples of 10 pHGGs were obtained from the Pathology Department of the Catholic University of the Sacred Heart and from Sapienza University in Rome. Three-micron-thick sections were stained with hematoxylin and eosin (H and E) for histology. Tumor diagnoses were confirmed by consensus of three neuropathologists (Felice Giangaspero, Manila Antonelli and Marco Gessi) based on World Health Organization (WHO) criteria [42]. 

### 4.3. Notch1 and Notch2 Immunohistochemistry

Immunohistochemical studies were performed using anti-activated Notch1 (#ab8925, Abcam, Cambridge, UK) and anti-Notch2 antibody (#HPA048743, Atlas Antibodies, Sigma Aldrich, Saint Louis, MI, USA). Paraffin-embedded slices of adult normal brain tissue purchased from the Biochain Institute (Newark, CA, USA) and of four no autopsy derived healthy brain tissues obtained from the Pathology Department of the Catholic University of the Sacred Heart and from Sapienza University in Rome were used as controls. The percentage of positive nuclei in each tumor sample and in the glial population of healthy brain tissues was calculated and results were scored as follows: 0 = nuclear positivity rate: 0–10%; 1 = nuclear positivity rate: 11–25%; 2 = nuclear positivity rate: 26–50%; 3 = nuclear positivity rate: 51–75%; 4 = nuclear positivity rate: 76–100% [43]. 

### 4.4. Cell Lines and Treatments

Functional studies were performed with KNS42 cells purchased from the Japanese Collection of Research Bioresources Cell Bank. Pediatric low-grade glioma cell lines Res259 and Res186 were kindly provided by Prof. Chris Jones from the Institute of Cancer Research in London. KNS42, Res259 and Res186 cells were grown in DMEM/F12 medium and squamous cell carcinoma (SC-011) were grown in RPMI 1640 medium. All media were supplemented with 10% fetal bovine serum (FBS, Sigma Aldrich, Saint Louis, MI, USA), 2 mM l-glutamine (Gln, Sigma Aldrich, Saint Louis, MI, USA) and 100 units·mL^−1^ antibiotic solution (100 units·mL^−1^ penicillin and 10000 μg·mL^−1^ streptomycin). Cells were cultured at 37 °C in a humidified 5% CO_2_ atmosphere.

To assess the effects of the treatments described below, KNS42 cells were seeded into 6-well plates (2.5 × 10^5^ cells/well), harvested at the indicated post-treatment time points (24, 48, 72, 96 h), and subjected to the Trypan Blue exclusion assay, by counting the number of cells that did not take up Trypan Blue, to evaluate cell growth. Res259 and Res186 were seeded into 6-well plates (1.8 × 10^5^ cells/well), harvested after 96 h and subjected to the Trypan Blue exclusion assay.

Pharmacological inhibition of Notch2—KNS42 cells were incubated with GSI (Calbiochem, Merck KGaA, Darmstadt, Germany) at concentrations of 2.5, 5.0 and 10.0 μM and CTRL (0.1% DMSO), harvested after 96 h of exposure, and assayed. 

Silencing of Notch2—For the silencing of Notch2 four individual ON-TARGET PLUS siRNA (LQ-012235-00, Dharmacon, Lafayette, CO, USA) were transfected at 100 nM each, or by using the ON-TARGET PLUS SMART pool (code: L-012235-00, Dharmacon, Lafayette, CO, USA). The ON-TARGET PLUS SMART pool yielded the best knockdown efficiency, therefore it was used for experiments, at the concentration of 100 nM. Silencing negative control was performed using ON-TARGETplus Non-targeting Control Pool (cat. #D-001810-10, Dharmacon, Lafayette, CO, USA). All siRNA were transfected using HiPerFect (Qiagen Inc., Milano, Italy). Proliferation was assayed 96 h after transfection.

MicroRNA overexpression—For single-microRNA overexpression, cells were transfected with 20 nM of one of the following synthetic microRNAs: miR-107 (miRIDIAN microRNA code: C-300527-03, Dharmacon, Lafayette, CO, USA); miR-181c (miRIDIAN microRNA code: C-300556-03, Dharmacon, Lafayette, CO, USA); miR-29a-3p (mirVana miRNA mimic code: MC12499, Ambion-Life Technology, Thermo Scientific, Wilmington, MA, USA); or with negative control (miRIDIAN microRNA negative control code: CN-001000-01-05, Dharmacon, Lafayette, CO, USA). For triple microRNA overexpression treatments, we used miR-107, miR-181c and miR-29a-3p at equal concentrations to obtain a final concentration of 20 nM. All transfections were performed with HiPerFect transfection reagent (Qiagen Inc., Milano, Italy). Cells were assayed 48 h after transfection.

### 4.5. RNA Isolation and qRT-PCR

The isolation of total RNA from fresh-frozen pHGG tissue samples and KNS42 cells have been previously described [44]. For microRNA analysis, single assay qPCR for assessment of miR-107 (code: 002112), miR-181c (code: 000443) and miR-29a-3p (code: 000482) were performed using TaqMan Individual microRNA assays (Applied Biosystems, Waltham, MA, USA), as previously described [5]. MicroRNA expression levels were normalized to U6 small nuclear RNA (Thermo Scientific).

### 4.6. Western Blotting 

Western blotting assays were performed as previously described [45] using the following primary antibodies: anti-Notch2 (D76A6) XP #5732 (Cell Signaling, Danvers, MA, USA), anti-NICD2 SAB4502022 (Sigma-Aldrich, St. Louis, MI, USA), anti-Notch1 C-20 sc-6014 (Santa Cruz Biotechnology, Dallas, TX, USA), anti-Sp1 (1C6) sc-420X (Santa Cruz Biotechnology), anti-cleaved PARP G7341 (Promega, Madison, WI, USA), anti-PARP #9542 (Cell Signaling), anti-GAPDH ab-8245 (Abcam, Cambridge, UK) and anti-β-Actin (I-19) sc-1616 (Santa Cruz Biotechnology). Horseradish peroxidase-conjugated secondary antibodies (Santa Cruz Biotechnology) were used to detect immunoreactive bands and binding was visualized by enhanced chemiluminescence (Perkin Elmer, Waltham, MA, USA). ImageJ software was used to perform band densitometry. Protein levels are expressed as relative to the internal control (β-Actin and Sp1). Nucleus/cytoplasmic fractionation was conducted as previously described [46].

### 4.7. Immunofluorescence Studies

Immunofluorescence studies were conducted according to standard procedures, as described elsewhere [47]. Briefly, KNS42 cells were plated on glass coverslips and fixed with 4% paraformaldehyde (PFA) for 10 min at room temperature (RT). Fixed cultures were permeabilized and blocked for 30 min with 5% donkey serum (DS) and 0.1% Triton X-100 (Sigma-Aldrich, St. Louis, MI, USA) in phosphate buffered saline (PBS, Sigma-Aldrich, St. Louis, MI, USA). Cells were then incubated overnight with anti-Notch2 antibody (#HPA048743, Sigma-Aldrich, St. Louis, MI, USA). Secondary antibody conjugated with Alexa Fluor 594 (Thermo Fisher Scientific) was diluted 1:200 in PBS with 5% DS and incubated with the specimens for 1 h at RT. Nuclei were counterstained with Hoechst reagent. After washing, slides were mounted using anti-fade reagent (Dako Fluorescence Mounting Medium, Carpinteria, CA, USA). Images were acquired using a FV1200 MPE laser scanning confocal microscope (Olympus, Shinjuku, Tokyo, Japan) with a UPlanSAPO 20×/0.75 NA objective. Imaris 8.1 software (Bitplane, Zürich, Switzerland) was used for image processing.

### 4.8. Plasmid Construction and Luciferase Reporter Assays

miR-29a-3p binding site in 3′-UTR gene regions was identified by bioinformatics analysis using microRNA.org (http://www.microrna.org/microrna/home.do) [48,49]. Human Notch2 3′-UTR (Figure S5) was amplified by polymerase chain reaction (PCR). A region of the human Notch2 3′-UTR (Figure S5) containing the putative miR-29a-3p binding site was amplified by PCR using the primers: Notch2 3′-UTR forward (5′-CACTCGAGAGTCCACCTCCAGTGTAG-3′) and Notch2 3′-UTR reverse (5′-CAGCGGCCGCAGTCAATGGAATGCTTG-3′) and cloned into psiCheck-2-luciferase reporter vector between the XhoI and NotI sites. 250 ng of the empty psiCheck2 vector or the recombinant plasmid containing the human Notch2 3′-UTR were transiently co-transfected into KNS42 cells with 50 nM of miR-107, used as positive control, or miR-29a-3p or negative control microRNA using Lipofectamine™ 2000 (Invitrogen, Thermo Scientific, Waltham, MA, USA). Twenty-four hours after transfection, cells were harvested and subjected to the Firefly Luciferase Assay 2.0 (Biotium, Fremont, CA, USA). Cells were incubated in 24-well plates for 15 min at room temperature with 100 µL of 1× Passive Lysis Buffer. Subsequently, 20 µL of the lysate was tested with 100 µL of firefly and Renilla solution in a 96-well plate. Luciferase activity was detected with a luminometer (GLOMAX, Fitchburg, WI, USA). Results are expressed as the ratio of Renilla to Firefly luciferase activity. Reported values are means ± S.D. of values from at least three experiments, each performed in triplicate.

### 4.9. Statistical Analysis

Data reported in this paper are the means ± SD of at least three independent experiments each performed in triplicate. Unpaired *t*-test, Paired *t*-test, one-way ANOVA and two-way ANOVA were performed wherever appropriate using GraphPad Prism Software version 6.0 (GraphPad Prism, La Jolla, CA, USA), *p* values <0.05 were considered to be statistically significant.

## Supplementary Materials

Supplementary materials can be found at www.mdpi.com/1422-0067/18/12/2742/s1.
