# Supplementary material for: Loss of miR-107, miR-181c and miR-29a-3p Promote Activation of Notch2 Signaling in Pediatric High-Grade Gliomas (pHGGs)
Source: Int J Mol Sci. 2017 Dec 17;18(12):2742. doi: 10.3390/ijms18122742 (PMC5751342; doi:10.3390/ijms18122742)
Supplement: Supplementary file 1 [file ijms-18-02742-s001.pdf]

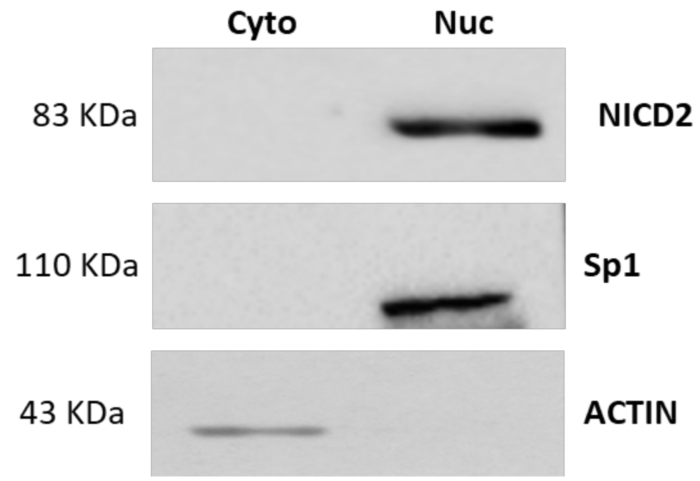

Figure S1. NICD2 localization in KNS42 after nucleus/cytoplasm fractionation. Western blot showing the subcellular localization of NICD2 in KNS42. NICD2 expression was analyzed both in the cytosolic (Cyto) and nuclear (Nuc) fractions. Sp1 and β-actin were used as loading controls and as markers for purity of Cyto and Nuc fractions, respectively.

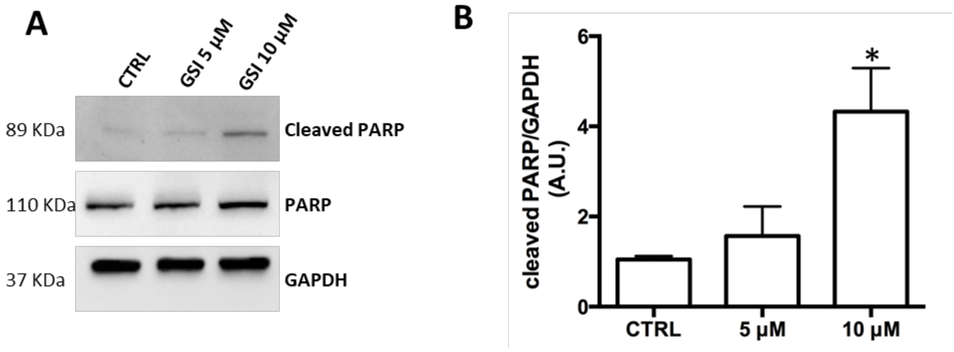

Figure S2. Cleaved PARP expression in KNS42 after GSI treatment. **(a)** Western blot showing the cleaved and the total form of PARP after 96h of GSI treatment at 5 and 10 μM. **(b)** Relative quantification of the level of expression of cleaved PARP after GSI treatment. GAPDH was used as loading control. *$p < 0.05$ *vs* CTRL.

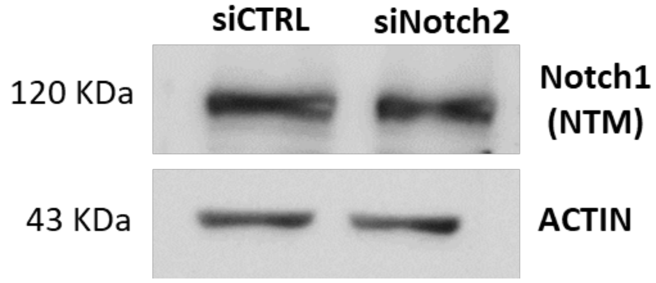

Figure S3. Notch1 expression in KNS42 cells after siRNA-mediated knockdown of Notch2. Western blot analysis of Notch1 levels 96 hours after Notch2 silencing in KNS42 cells.

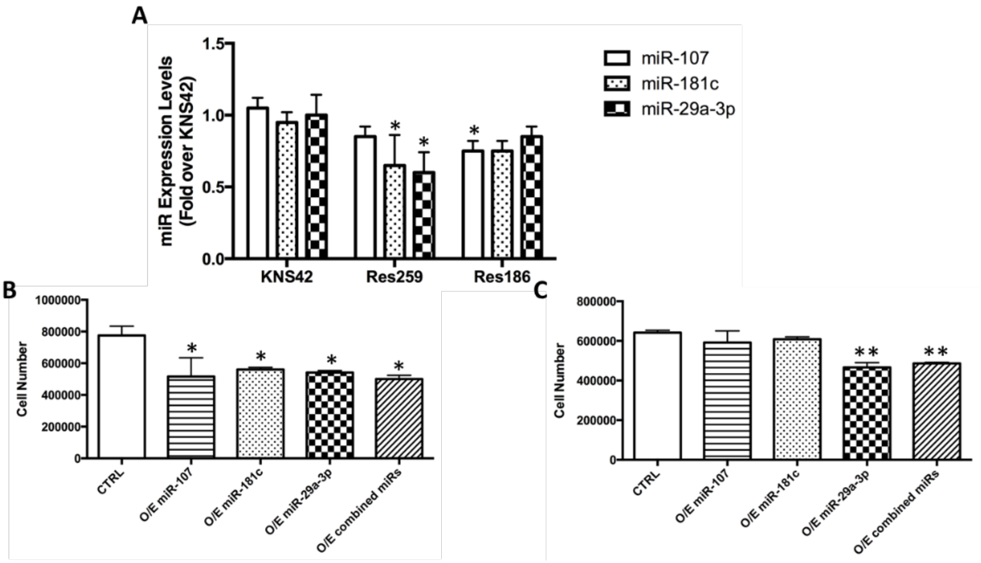

Figure S4. miR-107, miR-181c and miR-29a-3p inhibit glioma cell proliferation. **(a)** Single assay qPCR of miR-107, miR-181c, and miR-29a-3p expression in KNS42 cells versus Res259 and Res186, a grade II and a grade I pediatric glioma derived cell line, respectively. **(b-c)** Res259 **(b)** and Res186 **(c)** cells were transfected with the three microRNAs, separately and combined. Cell proliferation was evaluated 48 hours-post-transfection. **(a)** *p<0.05 *vs* KNS42. **(b-c)** *p<0.05, **p<0.01 *vs* CTRL.

gagtccacctccagtgtagagacataactgacttttgtaaatgctgctgaggaacaaatgaaggtcatccgggagagaaatgaagaaa
tctctggagccagcttctagaggtaggaaagagaagatgttcttattcagataatgcaagagaagcaattcgtcagtttcactgggtatc
tgcaaggcttattgattattctaatctaataagacaagtttgtggaaatgcaagatgaatacaagccttgggtccatgtttactctcttcta
tttggagaataagatggatgcttattgaagcccagacattcttgcagcttggactgcattttaagccctgcaggcttctgccatatccatg
agaagattctacactagcgtcctgttgggaattatgccctggaattctgcctgaattgacctacgcatctcctcctccttggacattcttttg
tcttcatt<u>**tggtgct**</u>tttggttttgcacctctccgtgattgtagccctaccagcatgttatagggcaagacctttgtgcttttgatcattctggc
ccatgaaagcaactttggtctcctttcccctcctgtcttcccggtatcccttggagtctcacaaggtttactttggtatggttctcagcacaa
acctttcaagtatgttgtttctttggaaaatggacatactgtattgtgttctcctgcatatatcattcctggagagagaaggggagaagaat
acttttcttcaacaaattttgggggcaggagatcccttcaagaggctgcaccttaatttttcttgtctgtgtgcaggtcttcatataaacttt
accaggaagaagggtgtgagtttgttgtttttctgtgtatgggcctggtcagtgtaaagttttatccttgatagtctagttactatgaccctc
cccactttttaaaaccagaaaaaggtttggaatgttggaatgaccaagagacaagttaactcgtgcaagagccagttacccacccaca
ggtcccccctacttcctgccaagcattccattgactgc

Figure S5. Region of the Notch2R 3′UTR cloned in the luciferase reporter vector. Underlined and bold is the binding site for miR-29a-3p.
